# Functional outcome after open reduction and internal fixation for symptomatic delayed union and nonunion after fracture clavicle: A series of 31 cases

**DOI:** 10.4103/0019-5413.33684

**Published:** 2007

**Authors:** John Mukhopadhaya, Swastik Shivapuri

**Affiliations:** Mukhopadhaya Orthopedic Clinic and Research Centre, Saidpur Road, Saidpur, Patna - 800 004, India

**Keywords:** Fracture clavicle, delayed union, nonunion, plate osteosynthesis, disabilities of the arm, shoulder and hand scores

## Abstract

**Background:**

Non-union after clavicular fractures can cause significant disability due to pain, impaired function of the shoulder joint and limitations of certain activities, especially in high-demand patients.

**Materials and Methods:**

31 patients (21 males and 10 females) of symptomatic delayed union or nonunion were treated operatively using plate osteosynthesis with bone grafting where required between January 1994 to September 2005. Out of the 31 patients, 14 cases were of delayed union (no evidence of union > six wks) and 17 cases were of nonunion (no union > three months). Fracture of the lateral one-third and open fractures of the clavicle were not included in the study. Bone grafting was done in 23 patients. The outcome was assessed with the American Academy of Orthopedic Surgeons (AAOS) disabilities of the arm, shoulder and hand (DASH) questionnaire.

**Results:**

The patients were followed-up for an average of 13 months (range six months to four years). All fractures united with in three months Most of the patients had their DASH scores in the range of 10 to 20, the average being 14.7 in our series. Functionally, this was very much acceptable.

**Conclusion:**

Open reduction and internal fixation with a plate in conjunction with an autogenous bone graft where required is a successful procedure with good outcome and most of the patients can return to a near normal level of function.

Despite the common occurrence of clavicular fractures, nonunion of a fracture of the clavicle is uncommon^1^–^4^ with a reported incidence of 0.9-4%.^5^–^12^ When it does occur, however, it can pose a difficult problem and can cause pain and impaired function of the shoulder girdle and upper limb. Rowe reported nonunion in 0.8% of fractures treated by closed methods.^4^ Even though displaced fractures of the clavicle often cannot be reduced and maintained in perfect position, cosmesis is acceptable and usually without functional disability. Nonunion is often asymptomatic. However, many patients have some functional disability which can be elicited if a detailed history is taken. They may have weakness, limitation of certain activities and sometimes the deformity may lead to pressure on the subclavian vessels or brachial plexus^13^–^17^ causing pain and troublesome neuralgia in the upper limb. Open reduction and internal fixation with or without bone grafting is indicated in these patients for correction of deformity to alleviate symptoms and improve function. In this series of 31 cases, we evaluated the functional outcome of open reduction and internal fixation for delayed union and nonunion after fracture clavicle with respect to relief from the pain and restoration of the daily activities.

## MATERIALS AND METHODS

31 patients of symptomatic delayed union or nonunion were treated by open reduction and internal fixation with or without bone grafting from January 1994 to September 2005. Twenty-one patients were male while 10 were females. Out of the 31 patients, 14 cases were of delayed union (no evidence of union at six weeks) and 17 cases were of nonunion (no evidence of union on clinical and radiological examination at three months).^4^,^18^–^20^ The average time of presentation following fracture was 14.2 weeks (range six weeks to seven years).

Only those patients who were symptomatic and their activities of daily living or their professional activities were affected, were included in this study. Fourteen of the 31 patients included in this study showed clinical mobility at the fracture site and inadequate callus formation on radiographic examination at the end of six weeks with significant morbidity due to pain and inability to use their limb to normal function. Such patients were classified as symptomatic patients with delayed union.

Weakness of the ipsilateral shoulder and functional limitations were seen in all the cases. Pain at the fracture site was present in 19 (61.3%) patients. Neuralgia in the C_7_ and C_8_ nerve root distribution was seen in six patients. Fracture of the lateral one-third and open fractures of the clavicle were not included in the study. We used dynamic compression plate (DCP) (n=18), low contact dynamic compression plate (LC-DCP) (n=7), reconstruction plates (n=2) and locking compression plates (LCP) (n=4). Bone grafting was performed in 23 patients [Table 1]. The patients were followed up for an average of 13 months (range six months to four years).

**Table 1:** Showing clinical details of the patients

Case No.	Age (years)	Sex	Time of presentation (weeks)	Presenting complaints	Type	Ancillary procedure	Follow-up (months)	DASH (post-op)
1	19	M	10	Weakness, pain	D/U	Local callus as bone graft	28	11.29
2	50	F	156	Weakness, pain	N/U	Bone graft	36	22.58
3	39	M	24	Weakness, pain	N/U	Local callus as bone graft	12	08.06
4	38	F	36	Weakness, pain	N/U	Bone graft	24	09.68
5	25	M	10	Weakness, pain neuralgia C7-C8	D/U	Bone graft	24	12.90
6	12	M	156	Weakness	N/U	Strut bone graft	24	14.52
7	60	M	14	Weakness	N/U	Local callus as bone graft	13	08.87
8	35	M	24	Weakness, pain	N/U	Bone graft	48	09.68
9	25	M	14	Weakness, pain	N/U	Bone graft	6	11.29
10	24	M	10	Weakness, pain	D/U	Bone graft	6	11.29
11	25	M	232	Weakness, neuralgia C7-C8	N/U	Strut bone graft	36	19.35
12	43	M	10	Weakness	D/U	Bone graft	6	16.94
13	16	M	6	Weakness, pain	D/U	-	7	17.74
14	18	F	18	Weakness	N/U	Bone graft	6	13.71
15	36	M	11	Weakness, pain	D/U	Bone graft	20	13.71
16	8	F	78	Weakness	N/U	Bone graft	40	10.48
17	70	F	14	Weakness, pain	N/U	Bone graft	8	21.77
18	51	F	364	Weakness	N/U	Strut bone graft	15	29.03
19	20	M	6	Weakness, pain neuralgia C7-C8	D/U	-	8	12.10
20	18	M	6	Weakness, pain	D/U	Bone graft	6	10.48
21	46	M	14	Weakness, neuralgia C7-C8	N/U	Local callus as bone graft	6	27.42
22	45	M	6	Weakness, pain	D/U	Bone graft	6	16.13
23	28	F	6	Weakness, pain, Neuralgia C7-C8	D/U	-	12	12.10
24	28	F	8	Weakness, pain neuralgia C7-C8	D/U	Bone graft	8	11.29
25	45	F	16	Weakness, pain	N/U	Local callus as bone graft	9	20.97
26	20	F	20	Weakness	N/U	Bone graft	6	11.29
27	40	M	12	Weakness	N/U	Bone graft, aspiration of hematoma	6	12.10
28	48	M	8	Weakness, pain	D/U	Bone graft	6	19.35
29	60	M	8	Weakness	D/U	Bone graft	6	14.52
30	48	M	6	Weakness, pain	D/U	Bone graft	10	14.52
31	24	M	12	Weakness	N/U	Bone graft	8	10.48

M = Male, N/U = non Union, F = Female, D/U = Delayed union, DASH - Disabilities of the arm, shoulder and hand

### Surgical technique

Surgery was done under general anesthesia with patient in beach chair position. A curvilinear incision was made over the clavicle to expose the fracture. Extreme care was taken while elevating the periosteum. The fracture ends were mobilized with careful sharp dissection, freshened and fracture was reduced with care not to damage the adjacent structures. Oblique fractures were stabilized using a cross-screw and a neutralization plate. In transverse fractures axial compression was given using a DCP, LC-DCP or an LCP. Cancellous chip bone graft was used where there was inadequate callus formation. In 8 cases bone graft was not used. Five of them had callus at fracture site, hence was used as bone graft, while 3 cases were 6 weeks old fracture and intraoperatively it was felt that good stability at fracture site will be good enough to achieve fracture union. Iliac crest strut graft was used in three patients where a significant defect existed at the site of nonunion after freshening bone ends. This not only restored clavicular length, but also helped to offset the aggravated stresses on the internal fixation.^4^,^16^ Stability of the fixation was checked clinically. The wound was closed in layers and a chest-arm bandage was given.

Wound was inspected on the second, sixth and ninth postoperative days. From the fourth postoperative day pendulum exercises were started and collar-cuff sling was given thereafter. Stitches were removed on the 12^th^ postoperative day. Full range of movement was allowed once the pain subsided. Patients were then reviewed at 6 weeks, 12 weeks and subsequently at three months interval.

The postoperative evaluation was based on inquiry regarding the pain, functional recovery, assessment of mobility, strength and stability of the shoulder joint and radiological appearance of the fracture. The final results were assessed using the disabilities of the arm, shoulder and hand (DASH) score. It was taken care that the patient answered at least 28 questions of the DASH questionnaire.

### DASH scores

DASH outcome measure is a 30-item, self-report questionnaire designed to measure physical function and symptoms in patients with any or several musculoskeletal disorders of the upper limb. The questionnaire was designed to help describe the disability experienced by people with upper-limb disorders and also to monitor changes in symptoms and function over time. Testing has shown that the DASH performs well in both these roles.

The DASH outcome measure was jointly developed by the Institute for Work and Health and the American Academy of Orthopedic Surgeons (AAOS). It gives clinicians and researchers the advantage of having a single, reliable instrument that can be used to assess any or all joints in the upper extremity. More severely disabled individuals have a higher score on a scale of 0 to 100.

## RESULTS

All the fractures united within three months [Figures 1–3]. The three patients who had iliac crest strut grafting also showed satisfactory incorporation of the graft. There was no fixation failure in the series. We did not encounter any major complications. Case no. 27 developed postoperative hematoma. The hematoma was aspirated and sent for culture and sensitivity. There was no organism grown on the bacterial culture. However, antibiotics were given for five days while awaiting culture reports. The wound healed without any evidence to suggest infection. Hypertrophic scar or keloid developed in three other patients. Except for two patients, we were able to achieve full range of motion in all the cases. Out of the six patients presenting with neuralgia in C_7_ and C_8_ nerve distribution, five had complete relief and one patient (case no. 21) had partial relief. He continued to have paraesthesia in the C_7_ and C_8_ distribution.

**Figure 1:**
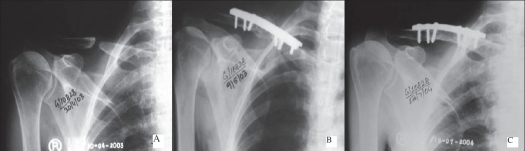
X-ray right shoulder anterior posterior view of a 51 years old female with 7 years old fracture clavicle. Showing. A) non-union of clavicle. B) fixation with DCP alongwith strut graft. C) 14 months old post operative X-rays

**Figure 1 D and E:**
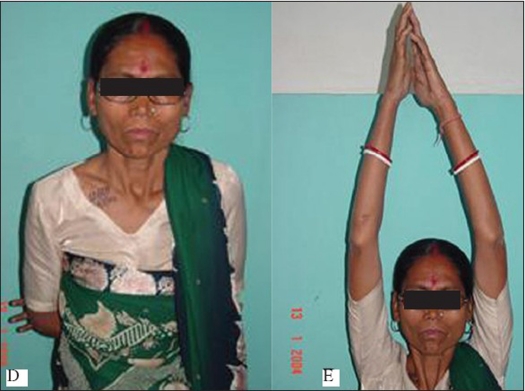
Clinical photograph of the same patient shows. D) Scar, internal rotation of shoulder. E) Abduction of shoulder

**Figure 2:**
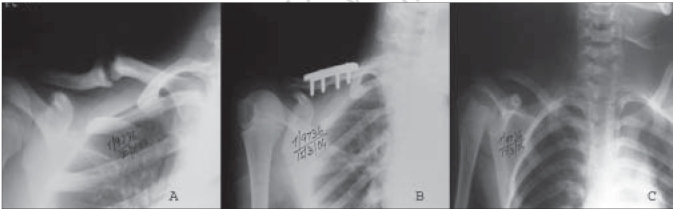
X-ray of shoulder antero- posterior view of an 8 years old female child shows. A) 1½ years old non-union fracture right clavicle. B) Post operative radiograph. C) Radiograph after removal of plate

**Figure 3:**
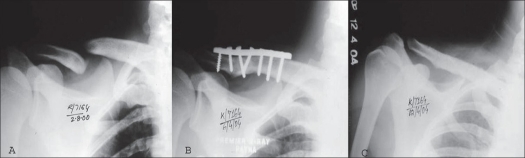
X-ray of shoulder antero- posterior view of a 35 years old male shows, A) 6 months old fracture clavicle. B) Union after DCP and bone grafting. C) After plate removal

Twenty-two patients observed their DASH scores in the range of 10 to 20. While four had less than 10 and 25 had more than 20. Mean DASH score of the patients in our series was 14.7. Functionally, this was very acceptable. In this series, 29 out of the 31 patients had full range of motion of the affected shoulder. Two had difficulty in abduction over 100°.

## DISCUSSION

The clavicle has an integral role not only in the mechanics of the pectoral girdle but also in the function of the upper extremity. Clavicle fractures rarely need operative fixation. Rowe suggested that the usual healing periods for fractures of the middle third of the clavicle were two weeks for infants, three weeks for children, four to six weeks for young adults and six weeks or more for older adults. Hence, we have taken absence of union at six weeks to define delayed union for clavicular fractures. The fractures which were operated at six weeks had frank mobility or sharp edges of bone pressing on skin which was a reason for not expecting the fracture to heal satisfactorily without intervention. We have considered nonunion in patients with clinical mobility at the fracture site and no radiological union after three months, double the time required for union as suggested by Rowe. Nonunion has been labeled after three months to 16 weeks in various studies.^17^–^19^ All our patients have demonstrable mobality clinically and showed no radiological evidence of healing. By symptomatic nonunion we mean that the patient has pain and weakness of the ipsilateral limb that hampers the activities of daily living.

Nonunion of a fracture of the clavicle although uncommon does occur and sometimes causes significant disability. Any osteosynthesis for a clavicular nonunion is subjected to complex forces from the muscles attached to it. This increases if there is shortening of the clavicle. The intramedullary pins or rods advocated in the treatment^3^,^4^,^21^–^24^ are technically difficult to insert because of the curvature of the clavicle. Moreover, they do not provide stability against the rotational forces.

Open reduction and plate osteosynthesis along with an autogenous bone graft where required, is a successful procedure with good functional outcome.^17^,^20^,^25^–^28^ Subclavian vessels and the brachial plexus lie beneath the middle third of the clavicle. Extreme care and meticulous dissection is required to prevent damage to these structures. Also, care must be taken to avoid damage to the pleura in the apical region of the lung. No force should be applied while drilling the holes. Power drills, preferably oscillating drills with sharp drill-bits should be used for the purpose. In general, the bone graft was given in patients who had an atrophic or oligotrophic type nonunion. Restoration of the length is necessary for good functional outcome. Cases who present very late with shortening of the clavicle or in severely comminuted fractures where surgical shortening is inevitable, a strut graft from the iliac crest should be used to restore the length of the clavicle and has given satisfactory results in our series.

We could achieve good functional outcome in most of our patients. The functional outcome by LC-DCP has been reported to be more satisfactory than the DCP^2^. However, in this study we achieved satisfactory results with both LC-DCP and DCP [Figures 1–3]. The results depended on the stability of the fixation, biological environment to achieve bone healing and careful surgical technique.

## CONCLUSION

Open reduction and internal fixation with a plate in conjunction with an autogenous bone graft, in selected cases, is a successful procedure with good outcome. With careful technique the complication rate is low and most patients can return to a near normal level of function. The high rate of success with plate fixation in this series and other series^17^,^20^,^25^–^28^ strongly support this statement.
